# N-Myc-induced metabolic rewiring creates novel therapeutic vulnerabilities in neuroblastoma

**DOI:** 10.1038/s41598-020-64040-1

**Published:** 2020-04-28

**Authors:** Britta Tjaden, Katharina Baum, Viktoria Marquardt, Mareike Simon, Marija Trajkovic-Arsic, Theresa Kouril, Bettina Siebers, Jan Lisec, Jens T. Siveke, Johannes H. Schulte, Uwe Benary, Marc Remke, Jana Wolf, Alexander Schramm

**Affiliations:** 1 Department of Medical Oncology, West German Cancer Center, University Hospital Essen, University of Duisburg-Essen, Essen, Germany; 2 Mathematical Modeling of Cellular Processes, Max-Delbrück-Center for Molecular Medicine, Berlin, Germany; 3 Division of Pediatric Neuro-Oncogenomics, German Cancer Consortium (DKTK) and German Cancer Research Center (DKFZ), Partner Site Essen / Düsseldorf; Department of Pediatric Oncology, Hematology and Clinical Immunology, Medical Faculty, University Hospital Düsseldorf; Department of Neuropathology, Medical Faculty, Heinrich-Heine-Universität Düsseldorf, Düsseldorf, Germany; 4 Division of Solid Tumor Translational Oncology, West German Cancer Center, Cniversity Hospital Essen, Essen, Germany and German Cancer Consortium (DKTK, partner site Essen), Essen, Germany; 5 Molecular Enzyme Technology and Biochemistry (MEB), Biofilm Centre, Faculty of Chemistry, University of Duisburg-Essen, Duisburg, Germany; 6 Federal Institute for Materials Research and Testing (BAM), Analytical Chemistry, Berlin, Germany; 7 Charité - Universitätsmedizin Berlin, Corporate Member of Freie Universität Berlin, Humboldt-Universität zu Berlin; Berlin Institute of Health (BIH), Berlin, Germany; Department of Pediatric Hematology/Oncology/Stem Cell Transplantation, Berlin, Germany; German Cancer Consortium (DKTK), Heidelberg, Germany, German Cancer Research Center (DKFZ), Heidelberg, Germany

**Keywords:** Cancer metabolism, Cancer metabolism, RNAi, RNAi

## Abstract

N-Myc is a transcription factor that is aberrantly expressed in many tumor types and is often correlated with poor patient prognosis. Recently, several lines of evidence pointed to the fact that oncogenic activation of Myc family proteins is concomitant with reprogramming of tumor cells to cope with an enhanced need for metabolites during cell growth. These adaptions are driven by the ability of Myc proteins to act as transcriptional amplifiers in a tissue-of-origin specific manner. Here, we describe the effects of N-Myc overexpression on metabolic reprogramming in neuroblastoma cells. Ectopic expression of N-Myc induced a glycolytic switch that was concomitant with enhanced sensitivity towards 2-deoxyglucose, an inhibitor of glycolysis. Moreover, global metabolic profiling revealed extensive alterations in the cellular metabolome resulting from overexpression of N-Myc. Limited supply with either of the two main carbon sources, glucose or glutamine, resulted in distinct shifts in steady-state metabolite levels and significant changes in glutathione metabolism. Interestingly, interference with glutamine-glutamate conversion preferentially blocked proliferation of N-Myc overexpressing cells, when glutamine levels were reduced. Thus, our study uncovered N-Myc induction and nutrient levels as important metabolic master switches in neuroblastoma cells and identified critical nodes that restrict tumor cell proliferation.

## Introduction

Myc proteins are encoded by a small family of proto-oncogenes consisting of *MYC*, *MYCN* and *MYCL*. In normal tissues, their expression is tightly regulated, while deregulation of *MYC* family members has been identified as a driving force in different cancer types. Since specific binding motifs, termed E-boxes, had been identified early on, Myc proteins were considered to be gene-specific transcription factors. This concept has been recently extended by different studies suggesting that deregulated Myc in tumors functions as a general transcriptional amplifier^1–3^. However, Myc-induced and tumor-specific mechanisms of target gene control on transcriptional level have only recently been addressed mechanistically^4^. The picture emerges that, at least in settings with vastly elevated Myc-levels, enhancer invasion by N-Myc and associated proteins contributes to tumor-specific N-Myc signatures. Moreover, the concept of Myc-mediated cell autonomous effects to boost tumor cell proliferation has been extended to include restriction of host immune reactions towards a tumor^5^. Although these efforts led to a better understanding of cell autonomous and cell non-autonomous regulatory circuits governed by oncogenic N-Myc functions, insights into mechanistic effects on the level of metabolic circuits is still largely lacking.

Deregulated Myc activity comes along with enhanced metabolic stress and increased sensitivity towards apoptosis due to a dependency on continuous supply with nutrients. Glutamine has been identified as a limiting factor for Myc-dependent cell growth and glutamine deprivation was preferentially inducing apoptosis in Myc-high cells^6^. In neuroblastoma, the most common solid tumor of childhood, elevated N-Myc levels are often found due to amplification of the coding gene, *MYCN*, which is correlated with poor overall survival of affected patients. N-Myc overexpression in the absence of *MYCN* amplification is not prognostic, pointing to the importance of additional genetic factors such as telomerase maintenance for determining disease outcome^7^. However, forced expression of N-Myc is sufficient to induce neuroblastoma in different model organisms including mice^8,9^ and zebrafish^10,11^ indicating a causative role for N-Myc expression in disease onset and maintenance. Ectopic N-Myc expression in neuroblastoma cells is accompanied with increased aggressiveness, but also a higher sensitivity towards drug-induced apoptosis *in vivo* and *in vitro*^12,13^. Interestingly, glutamine deprivation was reported to preferentially sensitize *MYCN*-amplified cells towards programmed cell death^14^. N-Myc also promoted expression of genes involved in glycolysis including lactate dehydrogenase (*LDH*) and hexokinase 2 (*HK2*) in neuroblastoma *in vitro*^15^ and *in vivo*^16^. The notion that depletion of LDHA inhibited *MYCN*-dependent tumorigenesis^17^ and that N-Myc cooperated with HIF1α^15^ suggested that N-Myc expression could also contribute to the Warburg effect. Thus, tumor-specific Myc deregulation directly affects tumor metabolism in multiple pathways.

At present, it is not clear how metabolic dependencies of tumors can be best exploited for therapeutic purposes. It is evident that metabolic features are remarkably flexible, especially those driven by Myc proteins. Moreover, tumor-type specific metabolic adaptations have been identified in Myc-driven tumors, exemplified by the Myc-mediated *de novo* synthesis of glutamine^18^. By contrast, Myc-driven liver tumors rather consume glutamine by a process termed glutaminolysis, which allows for fueling into the tricarboxylic acid cycle (TCA cycle) at the level of α-ketoglutarate by activation of glutaminase, another Myc-target^19^. *MYCN*-driven glutaminolysis has been recently suggested as a strategy to treat Myc-driven cancers^20^. Still, tumor cell-intrinsic response patterns as a consequence of *MYCN* activation under varying nutrient conditions largely remain to be identified. We thus set out to profile metabolic shifts in neuroblastoma cell lines with inducible N-Myc expression and correlate their phenotypic responses upon variations in the two most common carbon sources, glucose and glutamine.

## Materials and methods

### Cell culture and reagents

Neuroblastoma cell lines SHEP, SH-SY5Y, SK-N-AS and SK-N-SH were cultivated in RPMI1640 medium containing 10% fetal bovine serum (FBS) and antibiotics as described^21–23^. Protocols for generating inducible expression of a gene of interest have been described before^24^. In brief, cell lines were sequentially transfected with pcDNA6/TR, harboring the tetracycline repressor gene, and pT-REx-DEST30 (ThermoFisher/ Invitrogen) containing *MYCN* cDNA. Single cell clones were selected by limiting dilution in medium containing blasticidine and G418 (ThermoFisher/ Invitrogen). For all cell lines transfected to express N-Myc upon addition of tetracycline, the suffix “TR-MYCN” was added to distinguish them from the parental cells. N-Myc induction was realized by adding 1 μg tetracycline per ml medium. Cell lines were authenticated by STR genotyping prior and post transfections. All reagents used for cell culture were obtained from Gibco/ ThermoFisher. Absence of *Mycoplasma sp*. in cultivated cells was confirmed by performing PCR with Mycoplasma-specific primers (IDT).

### Metabolic activity, cell viability and cell proliferation assays

Metabolic activity was assessed using the 3-(4,5-dimethylthiazol-2-yl)-2,5-diphenyl tetrazolium bromide (MTT) assay for cells at the onset or in logarithmic growth phase. For monitoring cell viability upon inhibition of glutamine metabolism, automated cell handling in a 384 well format was used. Cell Titer Glo assays were performed using a Spark 10 M microplate reader (Tecan) as described^25^. For monitoring cell proliferation, a 3-(4,5-dimethylthiazol-2-yl)-2,5-diphenyltetrazolium bromide (MTT) assay was performed as described^26^.

### Protein analyses and antibodies

Either whole-cell protein extracts or fractionated cell extracts were separated by SDS-PAGE (10%) and subsequently used for western blotting. Immunoblot analysis was performed using primary antibodies targeting hexokinase II (1:500 dilution; #2106); hexokinase I (1:1000; #2804); N-Myc (1:1000; #9405); c-Myc (1:1000; #9402); PHGDH (1:1000, #13428, all from Cell Signaling Technology). To ensure equivalent protein loading, blots were stained with a primary antibody raised against β-actin (1:2000 dilution; #A5441, Sigma Aldrich Merck, Munich, Germany). Detection and visualization was achieved using HRP-conjugated goat anti-mouse or anti-rabbit secondary antibodies (GE Healthcare) and the ECL™ Prime Western Blotting Detection Reagent (GE Healthcare, #RPN2236), respectively. Data were analyzed using a FusionFX7 detection device (Vilber Lourmat).

### Real-time reverse transcriptase-PCR

Total RNA was isolated from cells using the High Pure RNA Isolation Kit (Roche, #11828665001), and cDNA synthesis was performed using the Transcriptor First Strand cDNA Synthesis Kit (Roche, #04379012001). Expression of target genes was monitored using a StepOnePlus™ Real-Time PCR System (Applied Biosystems) and Fast SYBR® Green Master Mix (Roche, #04913914001). Expression values were normalized to β-actin expression and the fold change was calculated using the ΔΔ − Ct method.

### Glycolysis flux assays

Glycolysis flux assays were performed in a Seahorse 96-well XF Cell Culture Microplate using a XF96 sensor cartridge according to the manufacturer’s instructions (Seahorse Bioscience, #102416-100). Briefly, 20,000 cells per well were allowed to grow overnight in a 37 °C, 5% CO2 incubator in pyruvate-free RPMI1640 (Gibco/ThermoFisher Scientific, #21875-034, #31870-025, #11879-020) with L-glutamine (Gibco/ThermoFisher Scientific, #25030-024) and varying concentrations of D-glucose (Gibco/ThermoFisher Scientific, #A2494001), 10% fetal bovine serum (Gibco/ThermoFisher Scientific #10270-106) and antibiotics. On the next day, medium was replaced with pyruvate- and glucose-free Seahorse XF Base Medium (#102353-100) supplemented with 2 mM glutamine according to the manufacturer’s protocol. The microplates were incubated at 37 °C for 45 min and then transferred to the microplate stage of a Seahorse XFe96 Extracellular Flux Analyzer to quantify the extracellular acidification rate (ECAR, in [mpH/min]) and the oxygen consumption rate (OCR, in [pmol/min]) using glucose concentrations as indicated. For each step, three separate ECAR and OCR readings using a mix and measure cycling protocol (3 min each) were recorded. Data for each experimental condition were calculated from a minimum of 10 technical replicates.

### Metabolic profiling

SHEP-TR-MYCN cells with and without induction of *MYCN* were incubated under varying glucose or glutamine concentrations. Upon harvesting, samples were prepared using the automated MicroLab STAR® system (Hamilton). To recover chemically diverse metabolites, proteins were precipitated with methanol under vigorous shaking for 2 min (Glen Mills GenoGrinder 2000) followed by centrifugation. The resulting extract was analyzed either by separate reverse phase (RP)/UPLC-MS/MS with positive ion mode electrospray ionization (ESI), RP/UPLC-MS/MS with negative ion mode ESI or HILIC/UPLC-MS/MS with negative ion mode ESI. The sample extracts were stored overnight under nitrogen before preparation for analysis. All methods utilized a Waters ACQUITY ultra-performance liquid chromatography (UPLC) and a Thermo Scientific Q-Exactive high resolution/accurate mass spectrometer interfaced with a heated electrospray ionization (HESI-II) source and Orbitrap mass analyzer operated at 35,000 mass resolution. Raw data was extracted, peak-identified and QC processed using hardware and software developed by Metabolon Inc..

### Statistical analysis

Statistical analyses of metabolic profiling data were performed using R, version 3.3.2 (available at cran.r-project.org). Prior to analyses, samples were normalized according to protein content determined by Coomassie G-250 based assays. For detailed procedures see Supplemental Methods. If not indicated otherwise, data are presented as mean and standard error of the mean (SEM).

## Results

### Metabolic reprogramming of SHEP cells depends on carbon source supply and N-Myc expression

**Figure 1:**
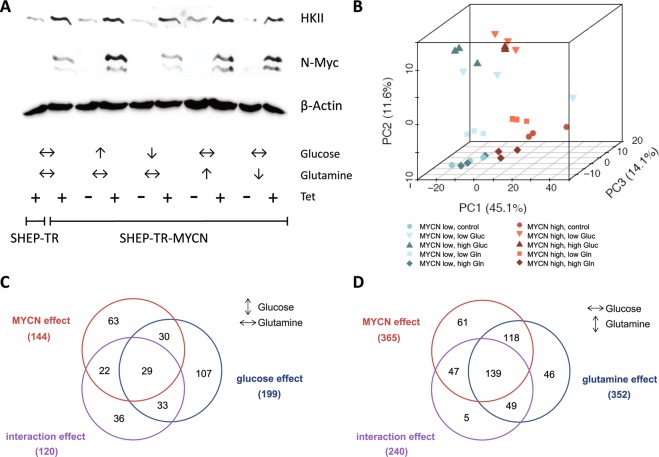
(**A**) Western blot analyses of hexokinase II (HKII) and N-Myc expression in SHEP-TR-MYCN cells. “+” and “−“ refer to induction of N-Myc expression by tetracycline (Tet). Arrows indicate high or low concentration of glucose and glutamine, respectively. Double arrows indicate concentrations of both carbon sources used for normal cell culture (cf. Table 1). (**B)** Principal component analysis (PCA) of metabolic profiling data using SHEP-TR-MYCN cells. In total, 495 metabolites present in at least 10 measurements were included. Each symbol denotes one sample. Blue symbols indicate conditions without N-Myc induction (“MYCN low”), while red symbols refer to samples with high N-Myc expression upon induction by Tet (“MYCN high”). Low and high levels of glucose and glutamine, respectively, were used as defined in Table 1. (**C)** Numbers of metabolites significantly affected by N-Myc, glucose abundance or with significant interaction effect according to bifactorial ANOVAs using sample groups with constant glutamine conditions and varying glucose levels (cf. Table 1). (**D)** Numbers of metabolites significantly affected by N-Myc, glutamine abundance or with significant interaction effect according to bifactorial ANOVAs using the sample groups with constant glucose conditions and varying glutamine levels (cf. Table 1).

We had previously described a correlation of *MYCN* with *HK2* mRNA expression in SHEP human neuroblastoma cells engineered to stably express N-Myc or to activate N-Myc only in the presence of tamoxifen^27^. To better understand the cellular and the metabolic response towards N-Myc induction and varying glucose /glutamine ratios, we here used SHEP cells with tightly controlled conditional upregulation of N-Myc in the presence of tetracycline (SHEP-TR-MYCN). Expression analyses confirmed both, N-Myc induction and concomitant hexokinase II protein (HKII) upregulation in these cells (Fig. 1A). SHEP-TR-MYCN cells were then cultivated with or without N-Myc induction and varying glucose and glutamine levels. Of note, N-Myc and HKII induction appeared to be independent of glucose and glutamine concentrations in the medium (Fig. 1A). We defined ten experimental groups that differed in N-Myc expression levels and nutrient supply (Table 1) to mimic different carbon source availability in the presence or absence of N-Myc. Metabolomic profiling by ultrahigh performance liquid chromatography-tandem mass spectrometry (UPLC-MS/MS) identified 499 compounds, of which 495 (99.2%) were present in at least one third of all samples. Upon normalization to protein content and imputation of missing values, principal component analysis (PCA) demonstrated separation of samples according to N-Myc expression. PCA also separately grouped samples cultivated at low or high concentrations of glucose or glutamine (Fig. 1B). We performed two condition-specific analyses of variance (ANOVAs) for each metabolite, one for constant glutamine conditions to examine the effect of glucose variation, and one for constant glucose conditions to examine the effect of glutamine variation. For each condition, we only analyzed metabolites which were present in at least one third of samples considered (Table 1). Induction of N-Myc was associated with consistent metabolic shifts, as 144/496 (29%, for constant glutamine) or 365/486 (75%, for constant glucose) of the metabolites were significantly altered (FDR < 0.05) between N-Myc high and N-Myc low expressing cells (Fig. 1C,D; Supplemental Figs. 1 and 2). A comparable number of metabolites were significantly affected when glucose (199/496, 40%) or glutamine levels (352/486, 73%) were varied. We then examined a possible interaction effect between N-Myc expression levels and glucose or glutamine supply. ANOVAs revealed a significant interaction between varying N-Myc expression and glucose concentrations affecting 24% of all metabolites (120/496 metabolites, FDR < 0.05) when only samples with constant glutamine concentrations were considered (Fig. 1C). An even higher number of metabolites (240/486, FDR < 0.05) had a significant interaction effect between N-Myc and glutamine when analyzing samples that had constant glucose concentrations (Fig. 1D). Classification of these metabolites according to “super pathways”^28^ was performed to uncover patterns according to metabolic categories. Here, metabolites classified in the category “energy metabolism” had the highest relative enrichment (Supplemental Figs. 1 and 2). Thus, global profiling suggested tight interaction of N-Myc and carbon source levels in metabolic reprogramming of SHEP cells, while HKII was confirmed as a N-Myc target independent of carbon source availability.

**Table 1:** Definition of groups for analyses of metabolite levels in SHEP-TR-MYCN cells with varying levels of carbon source supply as indicated (“Gluc”: glucose; “Gln”: glutamine).

Group	Cell line	Description	Gluc [g/L]	Gln [mM]	N-Myc	Gluc	Gln	Tet
1	SHEP-TR	without inducible N-Myc	2	2	/	↔	↔	+
2	SHEP-TR-MYCN	with inducible N-Myc	2	2	↑	↔	↔	+
3	SHEP-TR-MYCN	high Gluc, N-Myc low	4.5	2	↓	↑	↔	−
4	SHEP-TR-MYCN	low Gluc, N-Myc low	1	2	↓	↓	↔	−
5	SHEP-TR-MYCN	high Gln, N-Myc low	2	4	↓	↔	↑	−
6	SHEP-TR-MYCN	low Gln, N-Myc low	2	1	↓	↔	↓	−
7	SHEP-TR-MYCN	high Gluc, N-Myc high	4.5	2	↑	↑	↔	+
8	SHEP-TR-MYCN	low Gluc, N-Myc high	1	2	↑	↓	↔	+
9	SHEP-TR-MYCN	high Gln, N-Myc high	2	4	↑	↔	↑	+
10	SHEP-TR-MYCN	low Gln, N-Myc high	2	1	↑	↔	↓	+

N-Myc expression in these cells, but not in SHEP-TR cells, can be induced by addition of tetracycline (“Tet”). Additionally, definition of carbon source concentrations as “high”, “low” or unchanged (indicated by a double arrow) is explained here.

### N-Myc shifts the balance from oxidative phosphorylation to glycolysis

**Figure 2:**
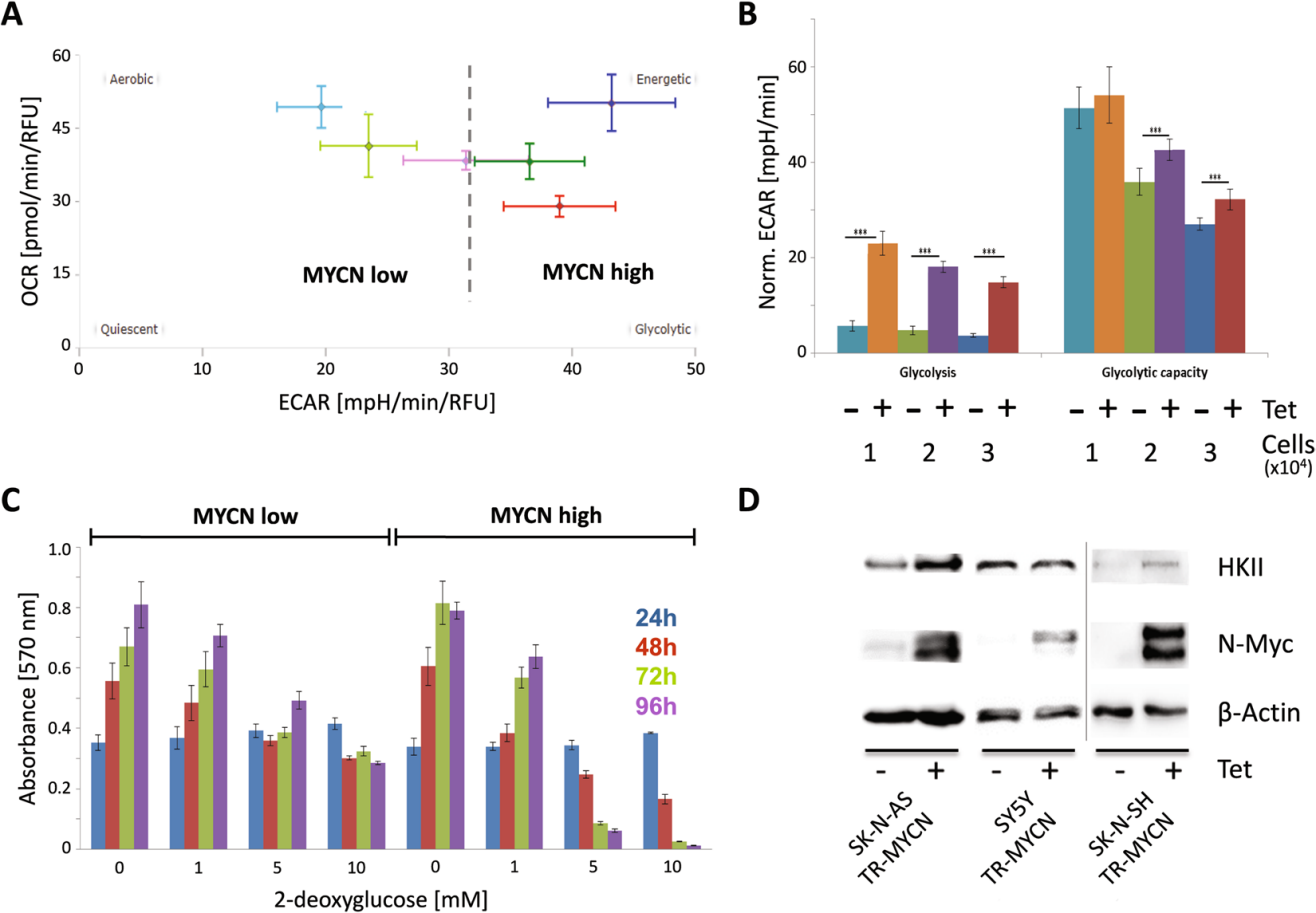
(**A**) Comparison of aerobic respiration and glycolysis indicated by the oxygen consumption rate (OCR) and extracellular acidification rate (ECAR) in SHEP-TR-MYCN cells with (“MYCN high”) and without induction of N-Myc (“MYCN low”). Colors indicate different glucose (Gluc) levels and N-Myc expression during cultivation and measurement; “MYCN low” cells are depicted in pink (1 g Gluc/l), light green (2 g Gluc/l) and light blue (4.5 g Gluc/l), while “MYCN high” cells are given in red (1 g Gluc/l), dark green (2 g Gluc/l) and dark blue (4.5 g Gluc/l). (**B)** Glycolysis and glycolytic capacity were monitored by measuring ECAR upon addition of external glucose and oligomycin in SHEP-TR-MYCN cells with and without induction of *MYCN* by tetracycline using cell numbers as indicated (“Norm. ECAR”: extracellular acidification rate normalized to 10,000 cells). The color code indicates different cell numbers and *MYCN* status, as 10^4^, 2 × 10^4^ or 3 × 10^4^ cells were used in the assay with and without induction of *MYCN* by tetracycline (“Tet”). Data were generated in triplicates in two independent experiments. Asterisks indicate a significance level p < 0.001. (**C)** SHEP-TR-MYCN cells were cultivated in the absence (“MYCN low”) or presence of tetracycline (“MYCN high”) and incubated with 2-deoxyglucose. At time points indicated, cell viability was recorded. Color code identifies samples analyzed at 24 h in blue, at 48 hours in red, at 72 hours in green and at 96 hours in purple. Data were generated in triplicates in two independent experiments. (**D)** Hexokinase II (HKII) and N-Myc protein expression in SK-N-AS TR-MYCN, SY5Y TR-MYCN and SK-N-SH TR-MYCN cells. “+” and “−“ refer to addition of tetracycline (Tet), which is regulating *MYCN* expression levels. The information presented here is derived from two experiments as indicated by the separating vertical line.

To examine if up-regulation of pacemaker enzymes of glycolysis including HKII were concomitant with a switch in energy harvest, we tested different scenarios in SHEP cells with varying N-Myc levels and glucose supply. In all conditions analyzed, we observed a shift towards preferential use of glycolysis upon *MYCN* induction as seen in higher ECAR levels (Fig. 2A). This effect was independent of glucose concentration (Fig. 2A) and cell number (Fig. 2B). Interestingly, N-Myc-induced upregulation of HKII did not result in altered steady state HK enzymatic activity. N-Myc low and N-Myc high cells also had comparable activity of other glycolytic proteins, glyceraldehyde-3-phosphate dehydrogenase (GAPDH), lactate dehydrogenase (LDH), triosephosphate isomerase (TIM) and pyruvate kinase (PK, Supplemental Fig. 3). Of note, we could confirm that c-Myc levels decrease upon N-Myc induction (Supplemental Fig. 3). Moreover, subcellular fractionation revealed no difference in HK activity for N-Myc low and N-Myc high cells in different cellular compartments (Supplemental Fig. 4). While siRNA-mediated knockdown of either HKI or HKII was tolerated regardless of N-Myc expression levels (Supplemental Fig. 5), blocking of glycolysis downstream of HK by using 2-deoxyglucose (2-DG) preferentially affected SHEP cells with high N-Myc levels (Fig. 2C). To check whether this effect could be recapitulated in other neuroblastoma cells, we ectopically expressed inducible N-Myc in three additional human neuroblastoma cell lines without *MYCN* gene amplification, SK-N-AS, SK-N-SH and SY5Y. Again, induction of N-Myc was accompanied by upregulation of HKII (Fig. 2D). N-Myc-mediated sensitization towards 2-DG was also observed for SK-N-AS cells, while SY5Y cells were intrinsically more sensitive to 2-DG regardless of *MYCN* expression levels (Supplemental Fig. 6). Thus, N-Myc overexpression induced a shift to preferential usage of glycolysis and also increased sensitivity towards inhibition of the glycolytic pathway in a cell line specific manner.

### N-Myc induces cell-type specific responses upon interference with different steps of glutamine biosynthesis

**Figure 3:**
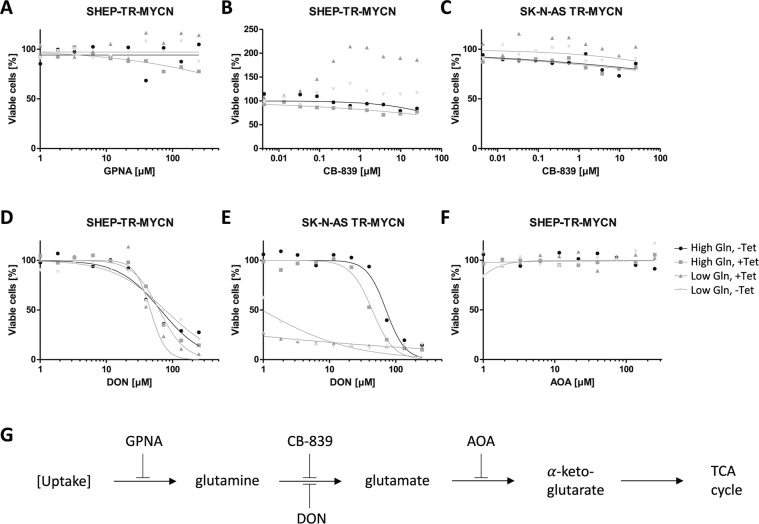
(**A–F**) Cell viability depending on small-molecule inhibitors of glutamine metabolism in cell lines indicated. Grey triangles indicate cells incubated in the presence of low Gln with high (dark grey) or low (light grey) N-Myc levels; rectangles and circles depict cells cultivated in the presence of high Gln with high (rectangles) or low levels (circles) of N-Myc. In experimental conditions indicated, high *MYCN* expression was induced by addition of tetracycline (“+ Tet”). (**G)** Scheme depicting the biochemical steps, in which the small molecule inhibitors of glutamine metabolism are supposed to act (AOA: aminoxyacetate; DON: 6-diazo-5-oxo-L-norleucine).

Cellular reprogramming by Myc proteins has been shown to affect glutamine metabolism, consistent with an increased demand of tumor cells for glutamine and metabolic adaptions to fluctuations in nutrient supply (reviewed in^29^). As for glucose, we observed a high number of significantly altered metabolites depending on *MYCN* expression levels when glutamine supply was varied. As indicated above, bifactorial ANOVAs revealed that 49% of all metabolites displayed a significant interaction effect between N-Myc induction and glutamine levels suggesting that these factors are intricately linked to metabolic reprogramming. As Myc proteins upregulate both, the import of glutamine and mitochondrial glutaminolysis, we aimed to investigate the cellular response to inhibition of glutamine metabolism upon *MYCN* induction. Blocking glutamine uptake by L-γ-Glutamyl-p-nitroanilide (GPNA) did not affect cell viability regardless of N-Myc expression levels (Fig. 3A). Paradoxically, inhibition of glutaminase by a small molecule inhibitor, CB-839, increased relative cell viability in SK-N-SH TR-MYCN and SHEP-TR-MYCN cells upon *MYCN* induction when glutamine supply was limited. However, CB-839 did not affect viability of SY5Y TR-MYCN and SK-N-AS TR-MYCN cells over a wide concentration range regardless of N-Myc and glutamine levels, while cell proliferation was reduced at high concentrations of CB-839 (Fig. 3B,C and Supplemental Fig. 7). Induction of *MYCN* highly sensitized SY5Y TR-MYCN and SK-N-AS TR-MYCN cells to low doses of the amino acid analogue DON (6-diazo-5-oxo-L-norleucine), which inhibits the conversion of glutamine to glutamate. Cell proliferation, but not cell viability of SHEP-TR-MYCN was affected by DON at concentration >10 µM, while cell viability of SK-N-SH TR-MYCN upon *MYCN* induction in the presence of DON was only reduced under low glutamine conditions (Fig. 3D,E and Supplemental Figs. 7 and 8). Blocking the link between glutaminolysis and the TCA cycle by inhibiting the conversion of glutamate to α-ketoglutarate using aminoxyacetate (AOA) did not affect either of the cell lines regardless of N-Myc or glutamine levels (shown exemplarily for SHEP-TR-MYCN in Fig. 3F). Thus, intervention with glutamine metabolism in neuroblastoma cells uncovered cell-type specific adaptations and metabolic dependencies modulated by *MYCN* expression and glutamine levels.

### N-Myc affects the entire pathway of glutathione biosynthesis, but exogenous glutathione does not restore viability of N-Myc high cells

**Figure 4:**
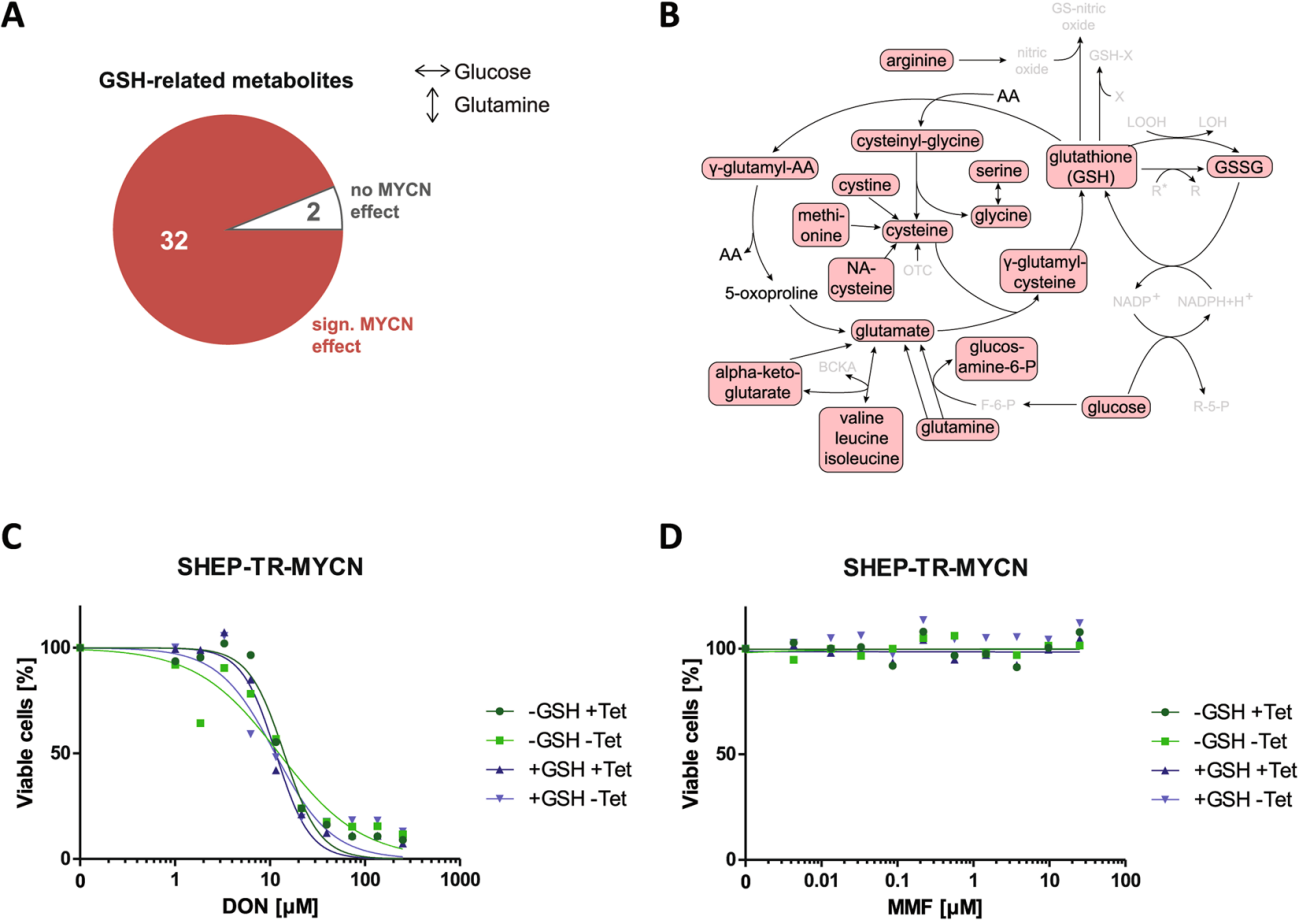
(**A**) Data presented in Fig. 1D were re-analyzed to visualize a significant N-Myc-mediated effect on glutathione (GSH) metabolism. In total, 32 out of 34 GSH-related metabolites are significantly affected by N-Myc levels according to ANOVA. (**B)** Main routes of the glutathione pathway (modified from^30^). Metabolites which are significantly affected by N-Myc for the sample groups under constant glucose and varying glutamine concentration in the medium are marked by red boxes. Interestingly, all significantly altered species are found to be decreased upon *MYCN* induction. Metabolites that have not been detected by UPLC are greyed (AA: amino acid, BCKA: branched chain keto acids, F-6-P: fructose-6-phosphate, GSSG: glutathione disulfide, LOOH: lipid hydroxyperoxide, LOH: lipid peroxyl radical, NA-cysteine: N-acetyl-cysteine, OTC: oxothiazolidine-4-carboxylate, R-5-P: ribose-5-phosphate, R, R*: radicals, X: xenobiotic). (**C,D**) Redox modulation by addition of GSH (100 mM) in the presence of varying concentrations of DON (**C**) or the ROS-inducer mono-methyl fumarate (MMF, **D**) in SHEP-TR-MYCN cells with or without induction of *MYCN* expression (“+Tet” and “−Tet”, respectively).

In our analysis of metabolome data, classification of known metabolites according to “super pathways”^28^ was also performed to uncover *MYCN*-induced patterns according to metabolic categories under varying glutamine concentrations. Significant overrepresentation of metabolites upon *MYCN* induction under these conditions was observed only for the group of “peptides” (Supplemental Fig. 9). Here, 26/26 di-peptides were significantly altered when cells with different N-Myc levels were compared. Detailed analyses of this group revealed that 12 of the di-peptides contained gamma-glutamyl residues and were linked to glutathione metabolism. Additional metabolites annotated as members of “glutathione metabolism”^30^ were also significantly altered by N-Myc levels independent of glutamine supply (32/34 metabolites, Fig. 4A); glutathione-associated metabolites were significantly over-represented among the *MYCN*-affected metabolites (p-value 0.003, Fisher’s exact test). Strikingly, almost all metabolites involved in glutathione metabolism, including glutamine, were found at significantly lower levels in N-Myc high cells compared to N-Myc low cells regardless of glutamine supply (Fig. 4B). While this suggested a limiting role of glutathione availability for N-Myc high cells, DON-mediated reduced viability of SHEP-TR-MYCN cells could not be rescued by exogenous glutathione supply (Fig. 4C). Additionally, increasing reactive oxygen species (ROS) by mono- or dimethyl fumarate (MMF, DMF) did also not affect cell viability upon *MYCN* induction and varying glutamine supply (Fig. 4D, Supplemental Fig. 10). These results indicate that *MYCN* induction causes a significant depletion of glutamine-related metabolites including glutathione, and that the resulting glutamine addiction cannot be attributed to modulation of ROS levels.

## Discussion

The wealth of data on plasticity of tumor cells suggests that blocking of single metabolic pathways may not be sufficient for long-term control of cancer growth. However, it is crucial to analyze oncogene-induced adaptations of metabolic pathways on a cellular level to understand mechanisms of metabolic plasticity contributing to development of therapy resistance in cancer. Still, tumor cell-centric assays are limited as they will miss interactions with the tumor microenvironment. A prime example is the notion that *KRAS* driven lung tumor cells are glutamine dependent *in vitro*, as they need glutamine-derived α-ketoglutarate to enter the TCA cycle, but are glutamine-independent *in vivo* due to fueling of the TCA cycle by glucose^31^. Expression of glutamine synthetase (GS) by cells of the tumor microenvironment might explain compensatory effects in cancer cells with reduced GS activity^32^. It has also been argued that routinely applied cell culture conditions will not appropriately mimic metabolic conditions found in human plasma^33^. Thus, experiments to model tumor cell responses to oncogenic activation *in vitro* have to be carefully designed. We here investigated the effects of ectopic *MYCN* expression in neuroblastoma cells lines in the presence of high or low supply with glucose or glutamine, respectively, as an approach to mimic both, oncogene-induced changes in metabolic patterns and response to altered carbon source supply. For metabolic profiling of these conditions, we chose SHEP neuroblastoma cells, in which we had previously noticed *MYCN*-induced *HK2* upregulation indicating metabolic reprogramming^16,27^. Using a newly adapted system for inducible *MYCN* expression, designated SHEP-TR-MYCN, we were able to demonstrate that HKII induction by N-Myc was independent of carbon source supply (Fig. 1A). Moreover, N-Myc dependent upregulation of HKII could be confirmed in other neuroblastoma cell lines (Fig. 2D). Metabolome analyses revealed that both, carbon source and N-Myc expression levels, cause significant and specific changes allowing for separation of these experimental groups by principal component analyses (Fig. 1B). Of note, we also observed significant interaction effects between N-Myc and carbon source levels (Fig. 1C,D) suggesting that N-Myc induced reprogramming also affects downstream metabolic processes under varying nutrient conditions. While our analyses uncovered that N-Myc mediated regulation of metabolites affects all pathways investigated, classification of N-Myc regulated metabolites confirmed enrichment for metabolites within the categories “energy metabolism” and a highly significant enrichment for metabolites involved in the glutathione pathway. We thus focused on analyses of two main pathways for energy harvest, glycolysis and glutamine metabolism, as the latter feeds also into glutathione biosynthesis. Indeed, we found a shift towards preferential use of glycolysis in *MYCN*-induced cells and an increased sensitivity towards glycolysis inhibition (Fig. 2). While this paper was in review, this was confirmed by results from Oliynyk *et al*. who showed that N-Myc increases the glycolytic and oxidative phosphorylation capacity of *MYCN*-amplified neuroblastoma cells. Moreover, they could demonstrate glutamine independence of mitochondrial respiration in *MYCN*-amplified neuroblastoma cells compared with non-*MYCN* amplified cells and that N-Myc high cells are especially vulnerable to inhibition of fatty acid oxidation^34^. We can conclude from these results that N-Myc reprograms neuroblastoma cells towards a highly energy producing and consuming phenotype. Glycolysis addiction in cancer cells with high levels of Myc has been described and linked to mTOR pathway activation^35^. Interestingly, we and others previously noticed an increased sensitivity of *MYCN*-dependent neuroblastoma towards mTOR inhibition^27,36^, suggesting that simultaneous blockade of glycolysis and mTOR would be an option to interfere with both, metabolism and survival pathways.

The addiction of *MYCN*-amplified neuroblastoma cells to glutamine availability has been described^14^. A recent report indicated higher glutamate/glutamine ratios in neuroblastoma compared to other cell types^37^. These findings suggested that neuroblastoma can be categorized as a “glutamine-consuming” rather than a “glutamine-producing” tumor type. Of note, c-Myc regulates glutamine metabolism, at least in part, by repressing miR-23a and miR-23b resulting in enhanced expression of their target gene, glutaminase^19^. Recent reports pointed to a possible feedback mechanism, as *MYC* translation itself is regulated by intracellular levels of glutamine-derived adenosine nucleotides via a sequence element in the 3′-UTR of the *MYC* mRNA^6^. In our model cell lines, this effect cannot be analyzed in the absence of endogenous regulator elements in the 3′UTR of the *MYCN* encoding vectors. The emerging picture, however, is far from clear: blocking glutamine uptake and its metabolic conversion to α-ketoglutarate was well tolerated in all conditions analyzed, indicating compensatory mechanisms. Paradoxical effects were observed for CB-839, an inhibitor of mitochondrial glutaminase (GLS1) that is currently tested in early clinical trials for treatment of several solid tumors multiple myeloma and acute myeloid leukemia^38^: while two of four neuroblastoma cell lines did not respond to CB-839, N-Myc expression combined with glutamine starvation enhanced metabolic activity of SK-N-SH and SHEP cells, however, this was not translating in increased cell proliferation (Supplemental Fig. 7). Viability of the latter two cell lines was not affected by DON, an irreversible glutamine-competitive inhibitor, at concentrations of up to 10 µM. However, limiting glutamine concentrations increased sensitivity towards DON in SY5Y and SKNAS cells, while N-Myc expression enhanced DON sensitivity 10-fold in SK-N-SH cells. Dissection of cellular responses on the level of the entire metabolome in SHEP cells revealed significant changes in glutathione-related metabolites upon variation of glutamine availability, which affected the vast majority of intermediates on the route from glutamine to GSH (Fig. 4A,B). We thus hypothesized that glutamine addiction of N-Myc high cells could be attributed to an increased sensitivity towards ROS production. Increased demand for ROS detoxification has been recognized as a vulnerability of cancer cells in the past years. The concept has also helped to understand the mechanisms of action for cytotoxic drugs including As_2_O_3_ and anthracyclines, which are effective at least in part by glutathione oxidation and increased ROS production, respectively^39^. However, sensitivity of N-Myc high cells to inhibition of glutamine metabolism could not be rescued by external glutathione supply. Moreover, N-Myc high cells were not significantly more sensitive to the ROS-inducing agent, dimethyl fumarate, which was described to suppress neuroblastoma cell proliferation^20^. Thus, preferential killing of N-Myc high cells in the presence of glutamine metabolism inhibitors and limited glutamine supply cannot be explained by increased consumption of glutathione and increased ROS sensitivity.

As mentioned above, strategies aiming to limit carbon source availability of inhibition of primary metabolic pathways have to take into account not only plasticity of tumor cells themselves but also that other cell types in the tumor microenvironment could serve as a carbon source *in vivo*^32^. Our data consistently show that responses to interference with primary metabolism are cell-type specific and that these cell-type specific differences largely contribute to metabolic plasticity reflecting inherent heterogeneity of our cell models. However, our results confirm interference with glucose and glutamine availability are possible avenues for exploiting metabolic dependencies of N-Myc high cells, which have to be further explored in *in vivo* models.

## Supplementary information

Supplementary Figures and Methods.

## References

[1] Lorenzin, F. *et al*. Different promoter affinities account for specificity in MYC-dependent gene regulation. *eLife* **5** (2016). 10.7554/eLife.15161 PMC4963202 27460974

[2] Walz S (2014). Activation and repression by oncogenic MYC shape tumour-specific gene expression profiles. Nature.

[3] Lin CY (2012). Transcriptional amplification in tumor cells with elevated c-Myc. Cell.

[4] Zeid R (2018). Enhancer invasion shapes MYCN-dependent transcriptional amplification in neuroblastoma. Nature genetics.

[5] Kortlever RM (2017). Myc Cooperates with Ras by Programming Inflammation and Immune Suppression. Cell.

[6] Dejure FR (2017). The MYC mRNA 3′-UTR couples RNA polymerase II function to glutamine and ribonucleotide levels. The EMBO journal.

[7] Peifer M (2015). Telomerase activation by genomic rearrangements in high-risk neuroblastoma. Nature.

[8] Weiss WA, Aldape K, Mohapatra G, Feuerstein BG, Bishop JM (1997). Targeted expression of MYCN causes neuroblastoma in transgenic mice. The EMBO journal.

[9] Althoff K (2015). A Cre-conditional MYCN-driven neuroblastoma mouse model as an improved tool for preclinical studies. Oncogene.

[10] Zhu S (2017). LMO1 Synergizes with MYCN to Promote Neuroblastoma Initiation and Metastasis. Cancer cell.

[11] Zhu S (2012). Activated ALK collaborates with MYCN in neuroblastoma pathogenesis. Cancer cell.

[12] Lutz W, Fulda S, Jeremias I, Debatin KM, Schwab M (1998). MycN and IFNgamma cooperate in apoptosis of human neuroblastoma cells. Oncogene.

[13] Fulda S, Lutz W, Schwab M, Debatin KM (1999). MycN sensitizes neuroblastoma cells for drug-induced apoptosis. Oncogene.

[14] Qing G (2012). ATF4 regulates MYC-mediated neuroblastoma cell death upon glutamine deprivation. Cancer cell.

[15] Qing G (2010). Combinatorial regulation of neuroblastoma tumor progression by N-Myc and hypoxia inducible factor HIF-1alpha. Cancer research.

[16] Schulte JH (2010). Deep sequencing reveals differential expression of microRNAs in favorable versus unfavorable neuroblastoma. Nucleic acids research.

[17] Dorneburg C (2018). LDHA in Neuroblastoma Is Associated with Poor Outcome and Its Depletion Decreases Neuroblastoma Growth Independent of Aerobic Glycolysis. Clinical cancer research: an official journal of the American Association for Cancer Research.

[18] Bott AJ (2015). Oncogenic Myc Induces Expression of Glutamine Synthetase through Promoter Demethylation. Cell metabolism.

[19] Gao P (2009). c-Myc suppression of miR-23a/b enhances mitochondrial glutaminase expression and glutamine metabolism. Nature.

[20] Wang T (2018). MYCN drives glutaminolysis in neuroblastoma and confers sensitivity to an ROS augmenting agent. Cell death & disease.

[21] Ackermann S (2011). Polo-like kinase 1 is a therapeutic target in high-risk neuroblastoma. Clinical cancer research: an official journal of the American Association for Cancer Research.

[22] Schulte JH (2008). Transcription factor AP2alpha (TFAP2a) regulates differentiation and proliferation of neuroblastoma cells. Cancer letters.

[23] Schulte JH (2005). Microarray analysis reveals differential gene expression patterns and regulation of single target genes contributing to the opposing phenotype of TrkA- and TrkB-expressing neuroblastomas. Oncogene.

[24] Pajtler KW (2014). Neuroblastoma in dialog with its stroma: NTRK1 is a regulator of cellular cross-talk with Schwann cells. Oncotarget.

[25] Stenzel K (2017). Alkoxyurea-Based Histone Deacetylase Inhibitors Increase Cisplatin Potency in Chemoresistant Cancer Cell Lines. Journal of medicinal chemistry.

[26] Schulte JH (2009). Lysine-specific demethylase 1 is strongly expressed in poorly differentiated neuroblastoma: implications for therapy. Cancer research.

[27] Schramm A (2013). Next-generation RNA sequencing reveals differential expression of MYCN target genes and suggests the mTOR pathway as a promising therapy target in MYCN-amplified neuroblastoma. International journal of cancer.

[28] Krumsiek J (2015). Gender-specific pathway differences in the human serum metabolome. *Metabolomics: Official journal of the Metabolomic*. Society.

[29] Dejure FR, Eilers M (2017). MYC and tumor metabolism. Chicken and egg. The EMBO journal.

[30] Wu G, Fang Y-Z, Yang S, Lupton JR, Turner ND (2004). Glutathione metabolism and its implications for health. The Journal of nutrition.

[31] Davidson SM (2016). Environment Impacts the Metabolic Dependencies of Ras-Driven Non-Small Cell Lung Cancer. Cell metabolism.

[32] Castegna, A. & Menga, A. Glutamine Synthetase: Localization Dictates Outcome. *Genes* **9** (2018). 10.3390/genes9020108 PMC5852604 29463059

[33] Cantor JR (2017). Physiologic Medium Rewires Cellular Metabolism and Reveals Uric Acid as an Endogenous Inhibitor of UMP Synthase. Cell.

[34] Oliynyk G (2019). MYCN-enhanced Oxidative and Glycolytic Metabolism Reveals Vulnerabilities for Targeting Neuroblastoma. iScience.

[35] Pusapati RV (2016). mTORC1-Dependent Metabolic Reprogramming Underlies Escape from Glycolysis Addiction in Cancer Cells. Cancer cell.

[36] Vaughan L (2016). Inhibition of mTOR-kinase destabilizes MYCN and is a potential therapy for MYCN-dependent tumors. Oncotarget.

[37] Kohe SE (2018). Metabolic profiling of the three neural derived embryonal pediatric tumors retinoblastoma, neuroblastoma and medulloblastoma, identifies distinct metabolic profiles. Oncotarget.

[38] Jacque N (2015). Targeting glutaminolysis has antileukemic activity in acute myeloid leukemia and synergizes with BCL-2 inhibition. Blood.

[39] Panieri E, Santoro MM (2016). ROS homeostasis and metabolism. A dangerous liason in cancer cells. Cell death & disease.

