# Supplementary material for: N-Myc-induced metabolic rewiring creates novel therapeutic vulnerabilities in neuroblastoma
Source: Sci Rep. 2020 Apr 28;10:7157. doi: 10.1038/s41598-020-64040-1 (PMC7188804; doi:10.1038/s41598-020-64040-1)
Supplement: Supplementary file 1 — Supplementary Figures and Methods. [file 41598_2020_64040_MOESM1_ESM.pdf]

Supplementary material for:

"N-Myc-induced metabolic rewiring creates novel therapeutic vulnerabilities in neuroblastoma"

Britta Tjaden[$], Katharina Baum[$], Viktoria Marquardt, Mareike Simon, Marija Trajkovic-Arsic, Theresa Kouril, Bettina Siebers, Jan Lisec, Jens T. Siveke, Johannes H. Schulte, Uwe Benary, Marc Remke, Jana Wolf, Alexander Schramm

[$] contributed equally

Corresponding author:

Prof. Dr. Alexander Schramm

Universitätsklinikum Essen (AöR)
Innere Klinik/ Tumorforschung
Molekulare Onkologie
Hufelandstraße 55
D-45147 Essen
Fon: +49 201 / 723-1630 Fax: +49 201/723 5616

e-mail: alexander.schramm@uk-essen.de

Contents

Supplemental figures with legends .... p. 2

Supplementary methods .... p. 12

Supplemental figures with legends

Suppl. Fig. 1

	MYCN effect	glucose effect	interaction effect
# metabolites (FDR<0.05)	144	199	120

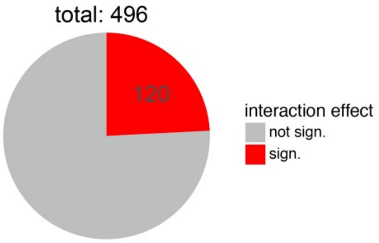

super pathway	# meta-bolites
Amino Acid (120)	26 (22%)
Carbohydrate (39)	12 (31%)
Cofactors & Vitamins (31)	9 (29%)
Energy (10)	4 (40%)
Lipid (190)	50 (26%)
Nucleotide (60)	14 (23%)
Peptide (28)	1 (4%)
Xenobiotics (18)	4 (22%)

Supplemental Figure 1: Metabolomic changes affected by N-Myc and media conditions when glucose concentrations were varied. Univariate, bifactorial ANOVA was applied for each of the 496 metabolites present in at least six samples. Numbers of significantly altered metabolites (FDR < 0.05 after multiple testing correction) that depend on N-Myc expression level (high or low; “MYCN effect”), medium condition (low glucose, normal glucose, high glucose; glucose effect) or their combined effect (interaction effect) in the 18 samples with varying glucose and constant glutamine abundances are given. Of 496 metabolites, 24% displayed a significant interaction effect (Venn diagram). Numbers of metabolites with significant interaction effect between N-Myc expression and glucose concentration in each category of the “super pathway” [1] classification and their percentage (from the number of metabolites measured in each category) are reported in the lower table.

Suppl. Fig. 2

	MYCN effect	glutamine effect	interaction effect
# metabolites (FDR<0.05)	365	352	240

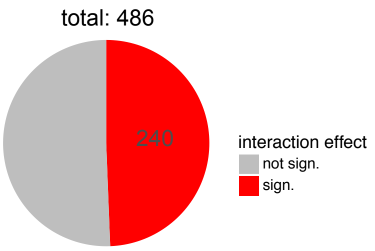

super pathway	# meta-bolites
Amino Acid (115)	62 (54%)
Carbohydrate (39)	22 (56%)
Cofactors & Vitamins (32)	16 (50%)
Energy (10)	8 (80%)
Lipid (185)	89 (48%)
Nucleotide (61)	29 (48%)
Peptide (26)	10 (38%)
Xenobiotics (18)	4 (22%)

Supplemental Figure 2: Metabolomic changes affected by N-Myc and media conditions when glutamine concentrations were varied. Univariate, bifactorial ANOVA was applied for each of the 486 metabolites present in at least six samples. Numbers of significantly altered metabolites depending on N-Myc expression level (high or low; "MYCN effect"), medium condition (low glutamine, normal glutamine, high glutamine; glutamine effect) or their combined effect (interaction effect) in the 18 samples with varying glutamine and constant glucose abundances are given. Of 486 metabolites, 49% displayed a significant interaction effect (Venn diagram). The numbers of metabolites with significant interaction effect between N-Myc expression and glutamine concentration in each category of the "super pathway" [1] classification and their percentage (from the number of metabolites measured in each category) are reported in the lower table.

Suppl. Fig. 3

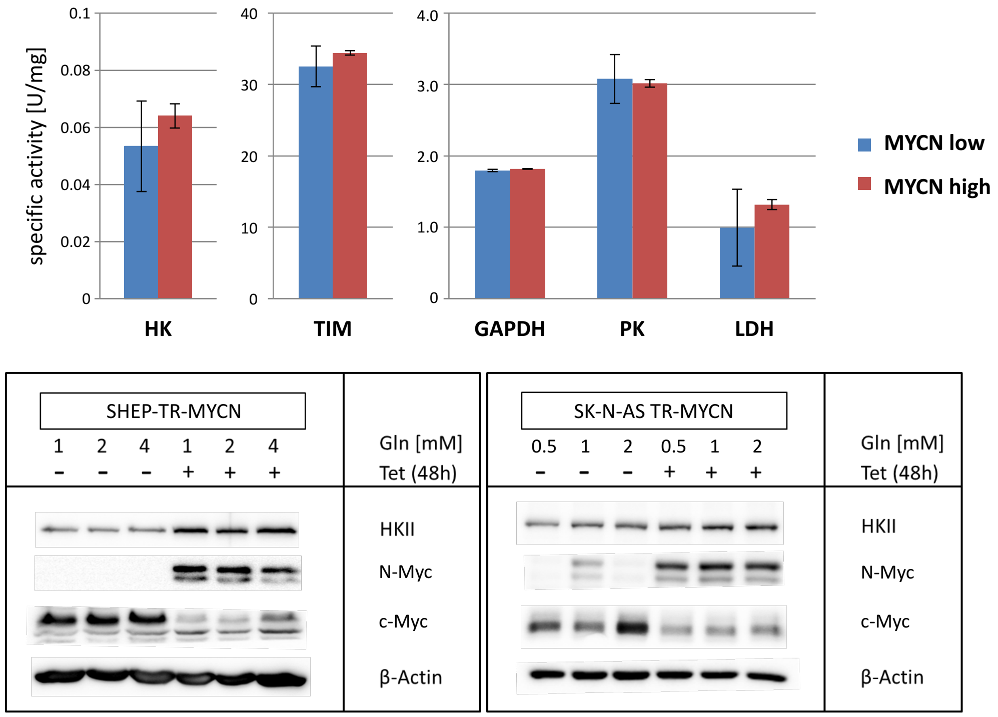

Supplemental Figure 3: Upper panel: Specific enzymatic activity in cell extracts from SHEP-TR-MYCN cells for the glycolytic enzymes hexokinase (HK), triosephosphate isomerase (TIM), glycerinealdehyde-3-phosphate dehydrogenase (GAPDH), pyruvate kinase (PK) and lactate dehydrogenase (LDH) was determined as described [2,3]. Enzymatic activities were determined in "MYCN low" (blue) and "MYCN high" (red) cells, when "MYCN high" was induced by addition of tetracycline. Lower panel: Western blot analyses of hexokinase II (HKII), N-Myc and c-Myc expression in SHEP-TR-MYCN and SK-N-AS TR-MYCN cells.

Suppl. Fig. 4

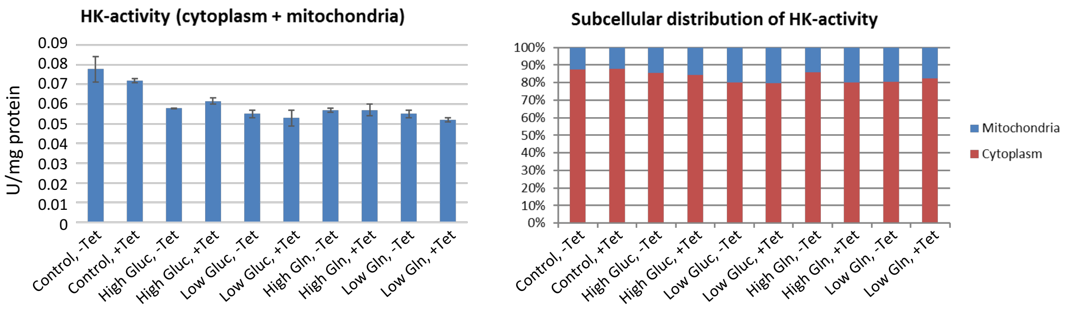

Supplemental Figure 4: Hexokinase (HK) activity was determined in SHEP-TR-MYCN cells under varying conditions as indicated. Left: HK activity in total cell extracts, right: Relative HK activity upon subcellular fractionation of cell extracts. No significant differences were observed between these conditions.

Suppl. Fig. 5

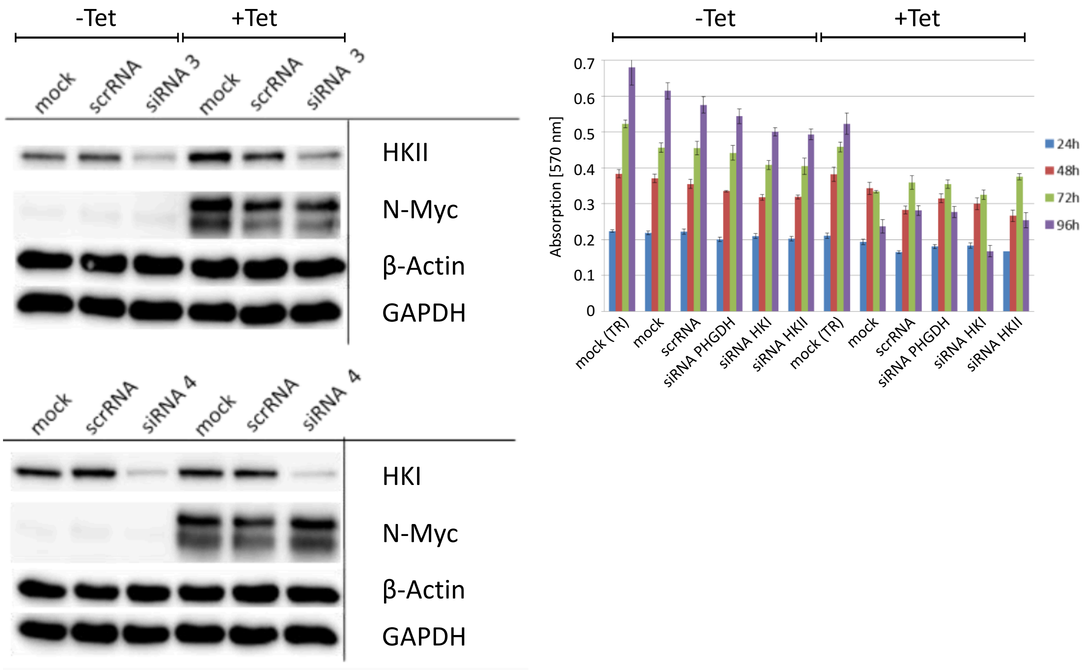

Supplemental Figure 5: Left: Isoform-specific knockdown of hexokinase I and II (HKI and HKII) in SHEP-TR-MYCN cells was achieved by specific siRNAs when compared to untransfected (mock) or scrambled RNA (scrRNA, siRNA 4 targets only HKI, while siRNA 3 is specific for HKII). Down-regulation of HKI and HKII was independent of *MYCN*-induction by tetracycline (Tet). Right: Neither downregulation of HKI nor HKII resulted in a significant reduction of cell viability compared to untransfected (mock) or scrambled RNA (scrRNA). Control-transfected SHEP-TR cells ("mock (TR)") and SHEP-TR-MYCN cells transfected with an siRNA targeting another metabolic enzyme, PHGDH, were used as additional controls.

Suppl. Fig. 6

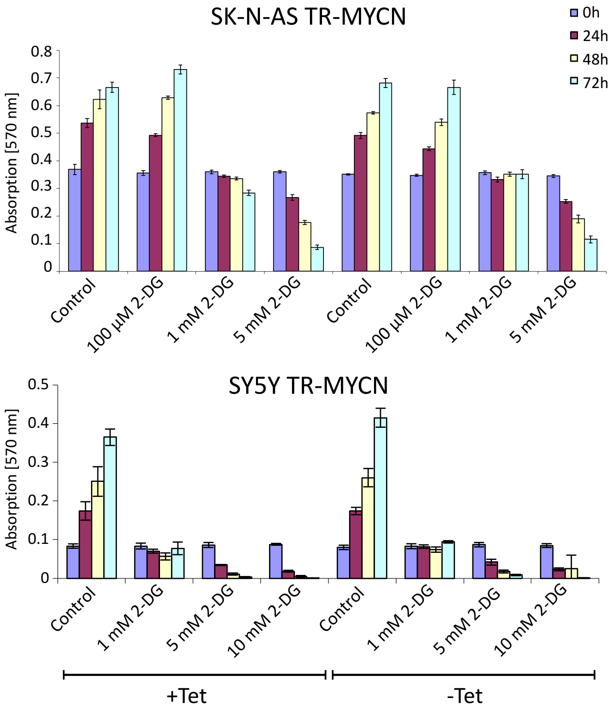

Supplemental Figure 6: Upper panel: SK-N-AS TR-MYCN cells were treated with 2-deoxyglucose (2-DG) for indicated time points (purple: t = 0; red: t = 24 h; yellow: t = 48 h; light blue: t = 72 h) and the cell viability was determined. Lower panel: SY5Y TR-MYCN cells were treated with 2-deoxyglucose and cell viability recorded as indicated above.

Suppl. Fig. 7

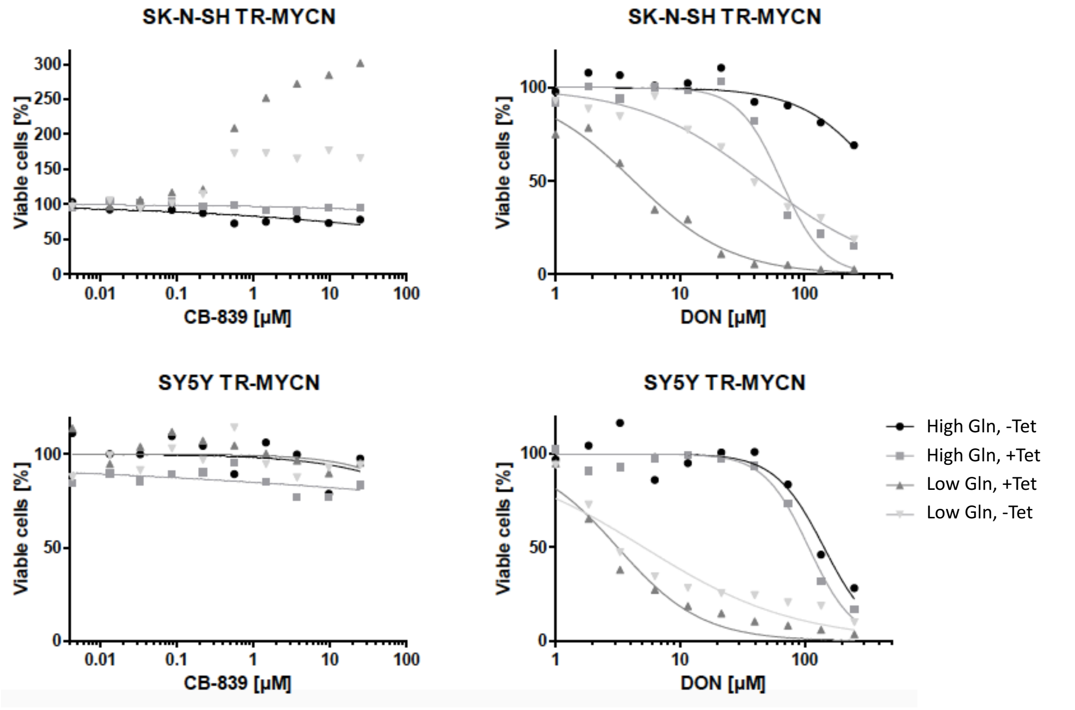

Supplemental Figure 7: Cell viability depending on a small-molecule inhibitors of glutamine (Gln) metabolism, CB-839 and DON, in cell lines indicated. Grey triangles indicate cells incubated in the presence of low Gln with high (dark grey) or low (light grey) N-Myc levels; rectangles and circles depict cells cultivated in the presence of high Gln with high (rectangles) or low levels (circles) of N-Myc.

Suppl. Fig. 8

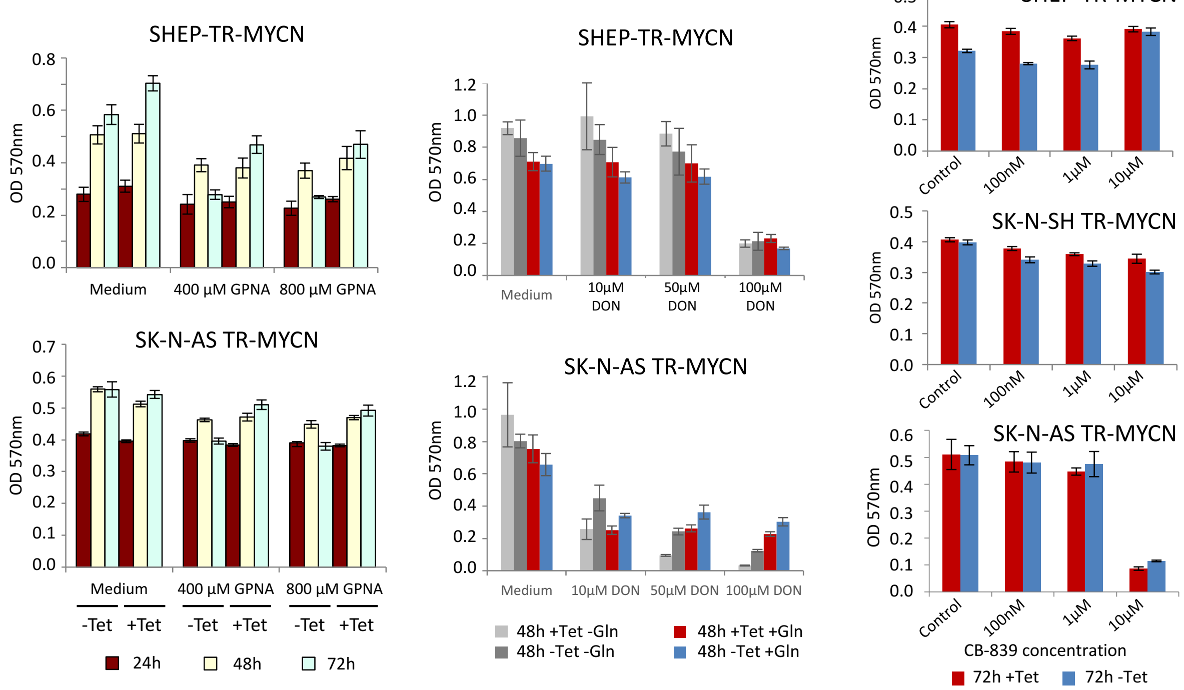

Supplemental Figure 8: Left: Proliferation of SHEP-TR-MYCN and SK-N-AS TR-MYCN cells in the presence of high concentration of the glutamine-uptake blocking agent, GPNA (red: t = 24 h; yellow: t = 48 h; light blue: t = 72 h, “+Tet” and “-Tet” indicate tetracycline-mediated *MYCN* induction and lack of *MYCN* induction, respectively). Middle: Proliferation of SHEP-TR-MYCN and SK-N-AS TR-MYCN cells in the presence of 4 mM glutamine (red and blue bars) or 1 mM glutamine (greyish bars). DON, which is an irreversible glutamine-competitive inhibitor, was added at t = 0 at concentrations indicated and proliferation was recorded 48 hours later. “+Tet” and “-Tet” indicate tetracycline-mediated *MYCN* induction and lack of *MYCN* induction, respectively. Right: Proliferation of SHEP-TR-MYCN, SK-N-SH TR-MYCN and SK-N-AS TR-MYCN with and without induction of *MYCN* (indicated by “+Tet” and “-Tet”, respectively). CB-839, which is a glutaminase inhibitor, was added at t = 0 at concentrations indicated and proliferation was recorded 72 hours later.

Suppl. Fig. 9

Significant MYCN effect for glutamine-varied conditions

super pathway	# meta-bolites
Amino Acid (115)	85 (74%)
Carbohydrate (39)	34 (87%)
Cofactors & Vitamins (32)	19 (59%)
Energy (10)	6 (60%)
Lipid (185)	139 (75%)
Nucleotide (61)	45 (74%)
Peptide (26)	26 (100%)*
Xenobiotics (18)	11 (61%)

* significantly over-represented

Supplemental Figure 9: Metabolites being significantly affected by N-Myc levels when glutamine concentrations were varied (according to an ANOVA, see also Fig. 1D) were classified according to categories of “super pathways” [1]. Numbers reflect the fraction of the total number of metabolites in each category. Upon correction for multiple testing, only the category “peptides” was found as significantly over-represented (corrected Fisher’s p-value < 0.05).

Suppl. Fig. 10

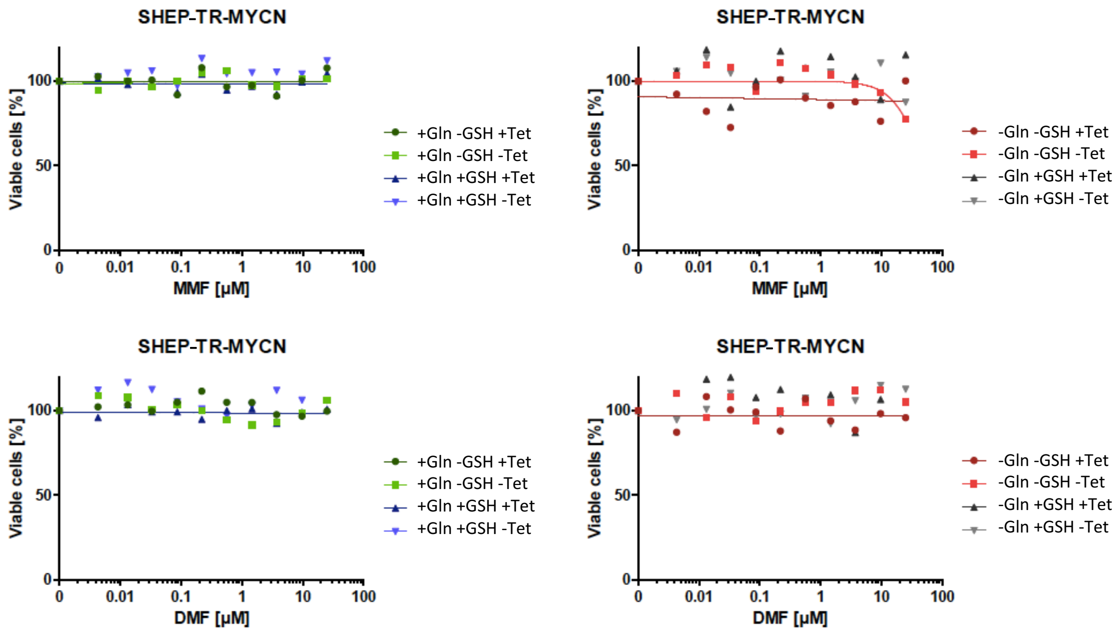

Supplemental Figure 10: Treatment of SHEP-TR-MYCN with mono- or dimethyl fumarate (MMF, DMF) in the presence of high (+Gln) and low (-Gln) glutamine medium concentrations, respectively. Additional variables were GSH concentration (100 µM= "+GSH", no external GSH= "-GSH") and high or low N-Myc expression induced by tetracycline (+Tet), which was absent in controls (-Tet).

Supplementary methods

Preparation of crude cell extracts for enzymatic assays

Protein lysates were generated applying ultrasound (five cycles, 30 s each in a Biodisruptor, Diagenode) to cells suspended in 50 mM HEPES/KOH, pH 7.5, containing 2 mM DTT and protease inhibitors (Complete, Roche). Lysates were centrifuged for 10 min, 700 g at 4°C to remove cell debris. A second centrifugation step (45 min, 21,000 g, 4°C) was applied to separate mitochondria cytoplasmic proteins, which were collected from the pellet or the supernatant, respectively.

Enzymatic assays

Apparent Km and Vmax values for LDH, HK, PK, TIM and GAPDH were determined using photometric assays based on NADH/NAD+ conversion as described earlier[2,3]. Specific activities were calculated as Units per mg protein. Glucose-6-phosphate dehydrogenase was used as an auxiliary enzyme to determine HK activity. Similarly, glycerol-3-phosphate dehydrogenase and LDH were added to determine TIM and PK activity, respectively. Reactions were started by addition of substrate or crude extracts.

Statistical analysis

As indicated in the main text, data pre-processing and analyses were performed in R, version 3.3.2 (https://cran.r-project.org).

Normalization, missing values and transformation of data from metabolic profiling

Samples were normalized to protein content as determined by Bradford assays. For each statistical analysis, metabolites with more than 2/3 of values being missing in the employed samples were discarded, i.e. 495/499 metabolites were used for the principal component analysis over all 30 samples. Additionally, 496/499 metabolites were included for the analyses of those 18 samples with varying glucose concentrations in the medium and 486/499 metabolites passed this filter for the analysis of the 18 samples with varying glutamine concentrations in the medium. The remaining missing values were imputed by the minimal intensity value for the metabolite across all measured samples. General conclusions were robust to different imputation strategies, e.g. either omitting any metabolites with NA values or imputing by a value drawn from a normal distribution around the lowest intensity for the metabolite (standard deviation: 1/6 of the mean), restricted to smaller values than the mean. Intensity values were square-root-transformed to ensure most similar variances between groups; this was determined by minimizing the number of metabolites with significances in Bartlett's test of homoscedasticity in a systematic Box-Cox-transformation analysis.

PCA

Principal component analysis was performed with scaling of the variance for each metabolite. Results were visualized using the R package scatterplot3d [4].

ANOVA

We performed univariate, bi-factorial analyses of variance (ANOVA) for each metabolite with the factors *MYCN*, medium (either "glucose concentration" or "glutamine concentration") and their interaction (MYCN:medium). The resulting p-values were corrected for multiple testing using the

method of Benjamini & Hochberg [5] over all factor effects together, but separately for either varying glucose or varying glutamine concentrations. We considered metabolites being significant towards one of the factors or their interaction for a corrected p-value < 0.05 (i.e. FDR < 0.05).

Annotation, overrepresentation analysis

We used the super-pathway annotation from [1] to sort the metabolites into 8 major categories. Significant over-representation of categories for interaction effects or *MYCN* effect were tested with Fisher's exact test, separately for glucose- or glutamine-varying conditions. We adjusted for multiple testing within each effect using Benjamini-Hochberg correction[5] and considered a category significantly over-represented if the corrected p-value was below 0.05.

Glutathione pathway characterization under glutamine-varying conditions

The annotation for metabolites of the class "glutathione metabolism" [1] was merged with that obtained from Wu et al. [6] resulting in 34 glutathione-associated metabolites among the 486 metabolites considered under glutamine-varying condition. 32 of the 34 glutathione-associated metabolites were significantly affected by *MYCN*; glutathione-associated metabolites were significantly over-represented among the *MYCN*-affected metabolites (p-value = 0.0034 of Fisher's exact test). For each of the three glutamine concentrations in the medium, the *MYCN* effect on each of the glutathione-associated metabolites were examined using a two-sided Welch's test comparing metabolite abundances for *MYCN* low and *MYCN* high cells. Glutathione-associated metabolites with Benjamini-Hochberg corrected p-values < 0.05 were considered significantly affected by *MYCN*.

References

1. Krumsiek, J. *et al.* Gender-specific pathway differences in the human serum metabolome. *Metabolomics : Official journal of the Metabolomic Society* **11,** 1815–1833 (2015).
2. Siebers, B., Wendisch, V. F. & Hensel, R. Carbohydrate metabolism in Thermoproteus tenax: in vivo utilization of the non-phosphorylative Entner-Doudoroff pathway and characterization of its first enzyme, glucose dehydrogenase. *Archives of microbiology* **168,** 120–127 (1997).
3. Schramm, A., Siebers, B., Tjaden, B., Brinkmann, H. & Hensel, R. Pyruvate kinase of the hyperthermophilic crenarchaeote Thermoproteus tenax: physiological role and phylogenetic aspects. *Journal of bacteriology* **182,** 2001–2009 (2000).
4. Ligges, U. & Mächler, M. Scatterplot3d - an R package for visualizing multivariat data, 1–20 (2003).
5. Benjamini, Y. & Hochberg, Y. Controlling the False Discovery Rate: A Practical and Powerful Approach to Multiple Testing. *Journal of the Royal Statistical Society. Series B,* 289–300 (1995).
6. Wu, G., Fang, Y.-Z., Yang, S., Lupton, J. R. & Turner, N. D. Glutathione metabolism and its implications for health. *The Journal of nutrition* **134,** 489–492 (2004).
